# Nutritional Solutions to Reduce Risks of Negative Health Impacts of Air Pollution

**DOI:** 10.3390/nu7125539

**Published:** 2015-12-10

**Authors:** Szabolcs Péter, Fernando Holguin, Lisa G. Wood, Jane E. Clougherty, Daniel Raederstorff, Magda Antal, Peter Weber, Manfred Eggersdorfer

**Affiliations:** 1 DSM Nutritional Products Ltd., Wurmisweg 576, Kaiseraugst 4303, Switzerland; daniel.raederstorff@dsm.com (D.R.); peter.weber@dsm.com (P.W.); manfred.eggersdorfer@dsm.com (M.E.); 2 Division of Allergy, Pulmonary and Critical Care Medicine, NW628 UPMC Montefiore Hospital, University of Pittsburgh Medical Center, 3459 Fifth Avenue, Pittsburgh, PA 15213, USA; holguinf@upmc.edu; 3 Centre for Asthma and Respiratory Diseases, University of Newcastle, Level 2, West Wing, HMRI Building, Kookaburra Crt, New Lambton Heights, NSW 2305, Australia; lisa.wood@newcastle.edu.au; 4 Department of Environmental and Occupational Health, University of Pittsburgh Graduate School of Public Health, Bridgeside Point I, 100 Technology Drive Room 350, Pittsburgh, PA 15219-3130, USA; jcloughe@pitt.edu; 5 Pannónia Street 66, Budapest 1133, Hungary; antalmagda@gmail.com

**Keywords:** air pollution, oxidative stress, inflammation, nutrients, vitamins, polyunsaturated fatty acids

## Abstract

Air pollution worldwide has been associated with cardiovascular and respiratory morbidity and mortality, particularly in urban settings with elevated concentrations of primary pollutants. Air pollution is a very complex mixture of primary and secondary gases and particles, and its potential to cause harm can depend on multiple factors—including physical and chemical characteristics of pollutants, which varies with fine-scale location (e.g., by proximity to local emission sources)—as well as local meteorology, topography, and population susceptibility. It has been hypothesized that the intake of anti-oxidant and anti-inflammatory nutrients may ameliorate various respiratory and cardiovascular effects of air pollution through reductions in oxidative stress and inflammation. To date, several studies have suggested that some harmful effects of air pollution may be modified by intake of essential micronutrients (such as B vitamins, and vitamins C, D, and E) and long-chain polyunsaturated fatty acids. Here, we review the existing literature related to the potential for nutrition to modify the health impacts of air pollution, and offer a framework for examining these interactions.

## 1. Introduction

Air pollution, including fine particulate matter (*i.e*., PM_2.5_) and gases, constitutes an environmental risk to human health and well-being. By estimation, around 80% of the world population lives in environments that exceed the air quality guideline (AQG) established by World Health Organization (WHO) [1,2]. In some regions, PM_2.5_ concentrations are reported to exceed air quality guidelines (AQG) (annual mean of 10 µg/m^3^) by several times [3]. While regulatory and environmental health efforts worldwide are working to reduce human exposures to air pollution, there may be related public health opportunities to reduce population susceptibility to air pollution.

Exposure to pollutants has been associated with increased rates of cardiovascular and respiratory morbidity and mortality, particularly in urban settings with elevated concentrations of primary air pollutants [4,5]. However, not every pollutant conveys the same risk and not everyone exposed is equally susceptible. In addition, there is no safe air pollution level at which adverse health effects are absent. These complexities have made it very challenging to legislate and develop adequate protection strategies.

Over the last decade, several studies have suggested that health impacts of some air pollutants (*i.e*., PM_2.5_) may be modified by individual intake of essential micronutrients and marine-derived long-chain polyunsaturated fatty acids (LC-PUFA, *i.e.*, fish oil) owing to their anti-oxidative and anti-inflammatory activities [6,7]. For instance, PM_2.5_-induced reduction in heart rate variability and alteration in oxidative status in humans may be ameliorated by these nutrients [8]. It has also been shown that antioxidant defenses are often impaired, and oxidative stress increased in asthma, which can also be exacerbated by air pollution [9,10]. Moreover, some clinical studies have indicated that the intake of antioxidants modulates inflammation, lung function and asthma symptoms [11].

Here, we summarize the research to date related to potential nutritional modification of air pollution health effects, towards better understanding the potential role for nutrition in modifying population susceptibility to pollution. We aim to provide a framework for future research towards reducing health impacts of air pollution worldwide, particularly for those populations that are most susceptible and/or most highly exposed [12].

## 2. Characteristics of Air Pollution

Understanding associations between air pollution and health is complicated in that air pollution is a tremendously complex mix of both gaseous and particulate compounds, varying across space and time by local emissions sources, meteorology, terrain, and other factors. Among the most-commonly studied air pollutants are fine particulates, often measured as PM_2.5_ (particles less than 2.5 microns in aerodynamic diameter, capable of moving through the bronchioles of the lung to the alveoli, hindering gas-blood exchange, or delivering metals, organic compounds, or other materials into the bloodstream). Gases associated with primary emissions (*i.e*., direct emissions from vehicular tailpipes, industry, or other combustion sources) are also well studied, and include nitrogen oxides (NO or NO_2_, collectively referred to as NO_x_), carbon dioxide (CO_2_), and sulfur dioxide. Together with lead (Pb) and ozone (O_3_, a secondary pollutant formed through photochemical reactions of combustion emissions in the presence of sunlight), these make up the six “criteria pollutants” regulated by the U.S. Environmental Protection Agency and other governmental bodies around the world [13,14].

The composition of air pollution varies across large regions—this movement of pollution across space is referred to as “long-range transport”—and within urban areas. Several recent models have been developed to capture air pollution variation across large spatial scales, such as across the continental U.S. [15,16]. It also varies tremendously within urban areas, where vehicular traffic, heating oil combustion, and other local sources lead to fine-scale spatial variation in population exposures. Recent studies in cities in multiple countries have documented and modeled this “intra-urban” (or, “within-city”) variation for use in health studies [17,18].

PM_2.5_ is a physical “gravimetric” measure, based on the total weight of fine particles that are airborne in any specific location at a moment in time. The chemical composition of those fine particles, however, varies drastically with the local source mix, meteorology, and photochemistry—even within a few meters of any given pollution source. Recent studies have documented variation in both elemental and organic composition of fine particles within urban areas [19,20,21], improving identification of local sources and better elucidating health effects linked to specific components or sources [22]. For example, a large study of air pollution across New York City linked patterns in airborne nickel—a component of PM_2.5_ previously associated with neurocognitive damage—with residual oil burning for heating in large buildings [23], leading to rapid policy changes regarding oil burning within the city.

Air pollution varies over time as well. Seasonal differences in pollution concentrations and composition, is perhaps most notable in elevated summer season concentrations of ozone. Similarly, air pollution concentrations and composition vary over the course of the day—given distinct “rush hours” of heavy traffic in most modern cities, and hourly variations in photochemical (sunlight-driven) chemical reactivity, meteorology, and the height of the atmospheric mixing layer.

There is growing evidence that the health effects of air pollution vary by chemical composition and by population susceptibility factors, such as socioeconomic position (SEP) [24,25], co-exposures such as tobacco smoke, and individual characteristics such as age or sex. As susceptibility factors like SEP and its associated stressors vary worldwide across urban areas [26], complex patterning in exposures and susceptibility together shape population responses to air pollution.

## 3. Impact of PM_2.5_ Exposure on Human Health

**Figure 1:**
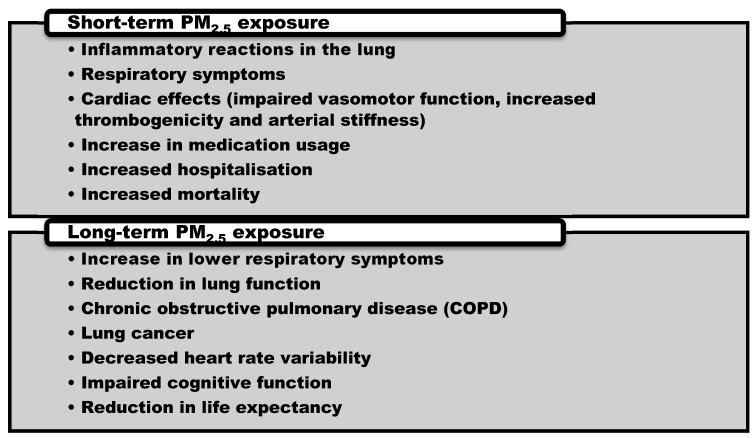
Health effects associated with short-term and long-term fine particulate matter (PM_2.5_) exposure [37].

It is widely shown that ambient air pollutants represent a significant risk factor to human health. Inhaled PM_2.5_ can reach the lung alveoli and induce local and systemic responses in the body, impacting cardiovascular and respiratory function [27,28]. Thus air pollution has been implicated in a range of illnesses worldwide, including respiratory and cardiovascular disease (CVD) [4,5], decreased heart rate variability (HRV) [29], impaired lung function [30,31], lung cancer [32,33], impaired cognitive function [34], increased mortality [35], and reduced life expectancy [36] (Figure 1).

**Figure 2:**
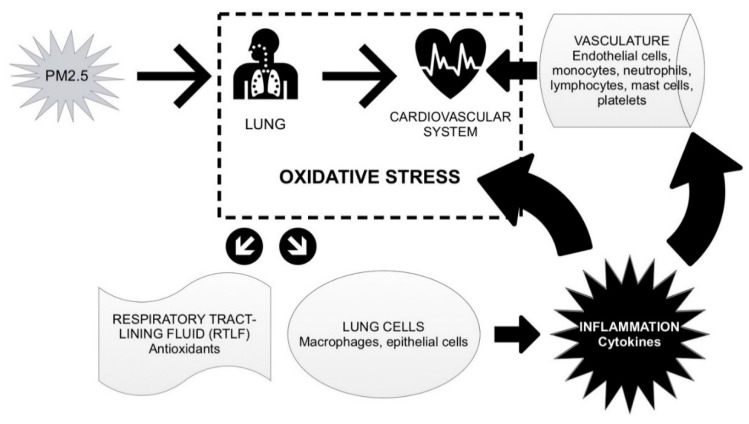
Possible biological pathways linking fine particulate matter (PM_2.5_) exposure with inflammatory and oxidative processes.

Air pollution effects on health may be mediated through a wide range of biological pathways, including inflammatory [38] and oxidative stress pathways [20,39]. However, there continues to be a considerable toxicological research effort to further characterize the mechanistic pathways of different types of PM and to detect chains of oxidative and proinflammatory events with relevance to the exacerbation of cardiorespiratory disease identified in epidemiological research (Figure 2) [40].

Some evidence suggests that PM_2.5_ may alter the function of the autonomic nervous system (ANS), which regulates cardiac function [27]. Thus, a dysfunction of the ANS leads to abnormalities in heart function, notably a decrease in heart rate variability (HRV). Decreases in HRV-related parameters are associated with increased cardiac morbidity and mortality. HRV declines with age and is considered as an early indicator of body stress and diseases. A study in Mexico City showed that exposure to increased concentration of PM_2.5_ was associated with cardiac autonomic dysfunction in elderly. The high frequency component of heart rate variability (HF-HRV) was significantly decreased for every 10 µg/m^3^ increase in same-day PM_2.5_. Moreover, individuals with hypertension were more susceptible to a reduction in HF-HRV induced by PM_2.5_ [41].

Acute exposure to pollutants is known to exacerbate symptoms of respiratory diseases such as asthma and chronic obstructive pulmonary disease (COPD) and increase the risk for cardiovascular complications such as stroke and myocardial infarction [27,42,43,44]. Chronic air pollution exposures have been associated with impaired lung growth in children and steeper decline in adults and with subclinical atherosclerotic disease, such as intima-medial thickening [31,45,46,47]. In addition to particle size, chemical composition and location, there are multiple host factors, such as age, genetic polymorphisms, clinical and subclinical disease, and co-exposures, which may modify individual susceptibility to air pollution.

Although the U.S. Environmental Protection Agency (EPA) and governmental agencies in other countries regulate a subset of air pollutants and have ambient quality metrics to protect public health, adverse health effects still occur within these “safe standards”. Results from some studies illustrate that there are temporal and spatial complexities, associated with air pollution and health effects that are not considered within metrics used by air quality regulations. For example, short-term exposure to traffic-related air pollution (in cities were pollutants are regulated below ambient standards), can be associated with significant airway physiological and inflammatory changes. Several studies examined the effects of short-term changes in air pollution on acute exacerbation of asthma and other diseases. Many of these were performed using times-series or case-crossover designs, examining acute hospitalizations or emergency department visits as a function of daily pollution concentrations. McCreanor *et al*. instructed healthy controls and patients with mild to moderate asthma to walk a 2 h prescribed route through Oxford Street in London (high exposure) or through Hyde Park (low exposure) on alternate days [48]. As expected, compared to Hyde Park, Oxford Street was associated with a higher PM_2.5_, ultrafine and elemental carbon exposure. In asthmatics, the Oxford walk led to a significant transient reduction in lung volumes and increased indices of airway inflammation and oxidation; in contrast, no associations were related with the Hyde Park walk. In a cohort study of nearly 5000 school age children from 13 California communities, in which proximity to a major road (defined as within 75 m) was associated with an increased risk for lifetime asthma [49], this risk decreased to background rates at 150–200 m from the major roads. It has also been observed that children residing in close proximity to major roads in Mexico City and in Ciudad Juarez, Mexico, had evidence of increased airway inflammation and reduced lung volumes [31,50].

Close proximity to major roadways—a commonly used indicator of exposure to primary traffic-related emissions—has also been associated with acute cardiovascular events. In a study of 691 survivors of myocardial infarction, recent exposure (within 1–2 h) to traffic was associated with a 2–3-fold increase in the OR for having acute myocardial infarction [51]. However, it must be noted that proximity to roadways is also correlated with noise exposure, socioeconomic position and other possible CVD risk factors as well. Interestingly, chronic particulate matter exposure has been linked, in a dose-dependent manner, with progression of intima-medial thickening [45]. Taken together, these results suggest that traffic-related pollution is associated with acute and chronic cardiovascular effects. While air pollution health effects may operate through a wide array of biological mechanisms, three predominant mechanisms for PM-mediated effects on CVD have been proposed: (a) Inhaled fine particles enter the blood stream leading to direct endothelial dysfunction and increased platelet aggregation (acute to chronic); (b) Particles cause pulmonary inflammation and oxidation, with a systemic “spill-over” effect, which may lead vascular dysfunction, hypercoagulability, insulin resistance, *etc.* (sub-acute to chronic); (c) Inhaled particles cause autonomic nervous system imbalance, leading to decrease heart rate variability and enhanced susceptibility to have cardiac arrhythmias (acute) [27].

Reduced pollution levels may readily translate into tangible health benefits, as suggested by two examples related to traffic-related air pollution reductions during two Olympic games. During the 1996 Olympic Games in Atlanta, USA, there was a significant reduction in traffic density, which led to reduced PM and nitrogen dioxide. In turn, the adjusted relative risk for acute asthma exacerbations requiring emergency department care was significantly reduced from baseline rates [52]. During the 2008 Beijing Olympics, a panel study of 201 healthy residents showed that, compared to the pre-Olympic period, peak flows increased in 78% of the study group during the games; unfortunately, a similar proportion showed a reduction in flows after the Olympics [53]. Similar associations were observed in a panel of 125 non-smoking healthy Beijing residents, in whom during the Olympics, several airway and systemic biomarkers of inflammation and oxidative stress, improved during the games, but returned to baseline at the conclusion of the event when restrictions in pollution emission eased [54].

## 4. Effect of Nutrients on Air Pollution-Induced Health Damage

A large body of evidence demonstrates the benefits of a healthy diet on promoting health and reducing the risk of chronic diseases. Individuals with a less nutritious diet, as assessed by the Overall Nutritional Quality Index (ONQI), are at higher risk of developing a number of chronic diseases [55]. Exposure to air pollution may further promote the risk and severity of these non-communicable disease, possibly through inflammatory and oxidative stress pathways [20,38,39,40].

The importance of nutrition for a healthy life has also been translated into dietary recommendations indicating that one should balance calorie intake with physical activity, prefer fruits, vegetables, fiber and fish and restrict salt, saturated fat and added sugar consumption. However, behavior change is very difficult to implement. Moreover, epidemiological studies have shown that specific nutrients such as omega-3 polyunsaturated fatty acids (PUFA) and an adequate intake of essential micronutrients can effectively reduce the risk of cardiovascular diseases and chronic diseases, through anti-oxidative and anti-inflammatory activities [56,57,58]. Several intervention studies in humans indicate that nutrients like omega-3 fatty acids and antioxidants may attenuate the damage induced by air pollution (Table 1) [6,59].

**Table 1:** Human clinical studies demonstrating the protective effects of nutrients in air pollutant exposure.

Reference	Design	Population	Sample Size	Air Pollutant	Nutrient Intervention	Objectives	Main Outcome Measures	Interpretation
**Mohsenin (1987) [60]**	Randomized, double-blinded, placebo-controlled	Healthy adults	*N* = 11	Nitrogen dioxide (NO_2_)	Vitamin C (4 × 500 mg/day)	To determine the effect of vitamin C on NO2-induced airway hyperresponsiveness in normal subjects	Lung mechanics and airway responsiveness to methacholine aerosol	Airway hyperresponsiveness induced by NO_2_ in normal subjects is completely prevented by pretreatment with ascorbic acid
**Romieu *et al.* (1998) [61]**	Randomized, double-blinded, placebo-controlled	Street workers	*N* = 47	Ozone (O_3_)	Vitamin C (650 mg/day), Vitamin E (75 mg/day), β-carotene (15 mg/day)	To evaluate whether acute effects of ozone on lung functions could be attenuated by antioxidant vitamin supplementation	Pulmonary function tests	Supplementation above the recommended allowance provided additional protection against the acute effect of high ozone exposure on lung functions
**Grievink *et al.* (1999) [62]**	Randomized, double-blinded, placebo-controlled	Bicyclists	*N* = 38	Ozone (O_3_)	Vitamin C (500 mg/day), Vitamin E (100 mg/day)	To investigate whether the acute effects of ozone on lung function could be modulated by antioxidant vitamin supplementation	Pulmonary function tests	Supplementation with the antioxidant vitamins C and E confers partial protection against the acute effects of ozone on FEV, and forced vital capacity in cyclists
**Trenga *et al.* (2001) [63]**	Double-blind crossover study	Adults with asthma	*N* = 17	Ozone (O_3_)	Vitamin C (500 mg), Vitamin E (400 IU)	To evaluate the effects of dietary antioxidants on ozone-induced bronchial hyperresponsiveness in adult subjects with asthma	Pulmonary function tests	Dietary supplementation with vitamins E and C benefits asthmatic adults who are exposed to air pollutants
**Romieu *et al.* (2002) [64]**	Randomized, double-blinded, placebo-controlled	Children with asthma	*N* = 158	Ozone (O_3_)	Vitamin C (250 mg/day), Vitamin E (50 mg/day)	To evaluate whether acute effects of ozone, nitrogen dioxide, and particulates with mass median diameter less than 10 µm could be attenuated by antioxidant vitamin supplementation	Pulmonary function tests	Supplementation with antioxidants modulated the impact of ozone exposure on the small airways of children with moderate to severe asthma
**Sienra-Monge *et al.* (2004) [65]**	Randomized, double-blinded, placebo-controlled	Children with asthma	*N* = 117	Ozone (O_3_)	Vitamin C (250 mg/day), Vitamin E (50 mg/day)	To investigate the impact of antioxidant supplementation on the nasal inflammatory response to ozone exposure in atopic asthmatic children	Nasal lavage	Vitamin C and E supplementation above the minimum dietary requirement in asthmatic children with a low intake of vitamin E provided some protection against the nasal acute inflammatory response to ozone
**Romieu *et al.* (2005) [8]**	Randomized double-blinded	Nursing home residents older than 60 years	*N* = 50	Particulate matter (PM)	Omega-3 fatty acid	To evaluate the effect of supplementation with omega-3 polyunsaturated fatty acids on the reduction of HRV associated with PM_2.5_ exposure	The association between HRV and 1 SD change in PM_2.5_ (8 µg/m^3^)	Supplementation with 2 g/day of fish oil prevented HRV decline related to PM_2.5_ exposure in the study population
**Romieu *et al.* (2008) [66]**	Randomized, double-blinded, controlled	Nursing home residents older than 60 years	*N* = 52	Particulate matter (PM)	Omega-3 fatty acid (2 g fish oil/day) Soy oil	To evaluate whether n-3 PUFA supplementation could protect against the cardiac alterations linked to PM exposure	Cu/Zn SOD activity, LPO products, GSH	Supplementation with n-3 PUFA modulated the adverse effects of PM (2.5)
**Riedl *et al.* (2009) [67]**	Placebo-controlled dose escalation	Healthy nonsmokers ≥18 years of age	*N* = 65	n/a	Oral sulforaphane doses contained in a standardized broccoli sprout homogenate	To investigate the *in vivo* effects of sulforaphane on the expression of glutathione-s-transferase M1 (GSTM1), glutathione-s-transferase P1 (GSTP1), NADPH quinone oxidoreductase (NQO1), and hemoxygenase-1 (HO-1) in the upper airway of human subjects	RNA expression for selected Phase II enzymes in nasal lavage cells	This study demonstrates the potential of antioxidant Phase II enzymes induction in the human airway as a strategy to reduce the inflammatory effects of oxidative stress
**Possamai *et al.* (2010) [68]**	Randomized, controlled	Subjects exposed to emissions from a coal electric-power plant	*N* = 80	Particulate matter (PM)	Vitamin C (500 mg/day), Vitamin E (800 mg/day)	To better understand the relations between PM exposure derived from a coal electric-power plant and the oxidative damage in subjects directly or indirectly exposed to airborne contamination	Biomarkers of oxidative stress	Antioxidant intervention was able to confer a protective effect of vitamins C and E against the oxidative insult associated with airborne contamination derived from coal burning of an electric-power plant
**Tong *et al.* (2012) [69]**	Randomized, double-blinded, controlled	Healthy middle-aged adults	*N* = 29	Particulate matter (PM)	Omega-3 fatty, acid (3 g fish oil/day), Olive oil (3 g/day)	To evaluate the efficacy of fish oil supplements in attenuating adverse cardiac effects of exposure to concentrated ambient fine and ultrafine particulate matter (CAP)	Heart rate variability (HRV) and electrocardiographic (ECG) repolarization changes. Plasma lipids changes	Omega-3 fatty acid supplements offer protection against the adverse cardiac and lipid effects associated with air pollution exposure
**Heber *et al.* (2014) [70]**	Controlled	Healthy subjects over age 18	*N* = 29	Diesel exhaust particle (DEP)	Sulforaphane (100 μmol)	To evaluate whether the administration of a standardized broccoli sprout extract could be used to suppress the nasal inflammatory response in human subjects challenged with 300 mg of an aqueous DEP suspension	White blood cell (WBC) counts	The study demonstrates the potential preventive and therapeutic potential of broccoli or broccoli sprouts rich in glucoraphanin for reducing the impact of particulate pollution on allergic disease and asthma
**Egner *et al.* (2014) [71]**	Randomized, placebo-controlled	Adults in good general health	*N* = 291	n/a	Glucoraphanin (600 µmol), Sulforaphane (40 µmol)	To evaluate the magnitude and duration of pharmacodynamic action of a broccoli sprout-derived beverage	Urinary excretion of the mercapturic acids of the pollutants, benzene, acrolein, and crotonaldehyde	Intervention with broccoli sprouts enhances the detoxication of some airborne pollutants and may provide a frugal means to attenuate their associated long-term health risks

Numerous studies have shown that a higher intake of omega-3 PUFA from fish oil can effectively lower the risk of cardiovascular disease through targeting risk factors such as hypertriglyceridemia and heart dysfunction [72,73,74]. However, only a few studies have addressed the effect of omega-3 PUFA on cardiac function in the presence of air pollution. In a randomized controlled trial in Mexico City, the effect of omega-3 PUFA on cardiovascular response to PM_2.5_ was evaluated in elderly residents of a nursing home [8]. The elderly were supplemented with 2 g/day of either fish oil or soy oil for five months, following a one-month pre-supplementation period. In the soy oil group, HF-HRV was decreased by 54% per one standard deviation (SD) increase in indoor PM_2.5_ (24 h average), while only a 7% decrease in HF-HRV was observed per one SD increase in PM_2.5_ after fish oil supplementation. Thus, omega-3 PUFA from fish oil prevented the negative impact of PM_2.5_ on HRV in elderly adults [8].

The effects of omega-3 PUFA supplementation on the oxidative response induced by exposure to PM_2.5_ was also investigated in this elderly population from Mexico City [66]. Inhaled pollutants, such as PM_2.5_, react with nonenzymatic antioxidant constituents of the respiratory tract lining fluid (RTLF) including reduced glutathione (GSH) and enzymatic antioxidants, such as superoxide dismutase (SOD). Therefore, antioxidant composition of the RTLF might be an important determinant of individual susceptibility to air pollutants [75]. If defenses are insufficient, the production of reactive oxygen species (ROS) is increased and oxidants may react with organic molecules. The ability of the lung to up-regulate protective ROS-scavenging systems, and also the synergy of endogenous and exogenous antioxidants, is key in the neutralization of free radicals in the respiratory passage [76]. Omega-3 PUFA intake from fish oil (2 g/day) for four months increased SOD activity by 49% and the level of GSH by 62%, and decreased lipoperoxidation (LPO) by 72%. Thus, omega-3 PUFA appear to modulate PM_2.5_ induced oxidative stress by increasing the activity of endogenous antioxidants [66]. Tong *et al.* [69] evaluated the effect of fish oil-derived omega-3 PUFA supplementation (3 g/day for four weeks) on acute sequential exposure of healthy middle aged individuals to either filtered air or concentrated ambient fine and ultrafine particles (CAP, mean mass concentration 278 µg/m^3^) for 2 h. The study concluded that omega-3 PUFA protected against the deleterious cardiac and lipid effects induced by acute exposure to particulate matter [69].

The B vitamins including vitamin B_2_, vitamin B_6_, vitamin B_12_ and folate are key cofactors and substrates of one carbon metabolism via the folate and methionine cycles. One carbon metabolism involves numerous methylation reactions, the biosynthesis of lipids, nucleotides and proteins. A perturbation of one carbon metabolism is associated with many diseases such as cardiovascular disease, neurological disease and cancer [77,78]. A study in elderly adults from the Boston area evaluated the effects of air pollution, gene polymorphisms in one carbon metabolism and dietary intake of methyl nutrients (folate, vitamin B_6_, vitamin B_12_ and methionine from food sources) on HRV [79]. For each 10 µg/m^3^ increase in ambient PM_2.5_ levels, 48 h before the HRV measurement, a 7% decrease in HRV was observed. Moreover, individuals carrying the methylenetetrahydrofolate reductase (MTHFR) C677T CT/TT genotypes, which are also at higher risk of CVD, had a significantly more pronounced decrease in HRV than those carrying the homozygous CC genotype. Finally, in individuals with a higher dietary intake of vitamin B_6_ (≥3.65 mg/day), vitamin B_12_ (≥11.1 µg/day), folate (≥495.8 µg/day) and methionine (≥1.88 mg/day), the negative effect of PM_2.5_ on HRV was prevented. Thus, the impact of PM_2.5_ on health can be modulated by genetic variations and dietary intake of micronutrients [79].

Vitamin E and vitamin C are respectively the key lipid-soluble and water-soluble antioxidants in humans. The effect of PM induced oxidative stress was evaluated in individuals exposed directly or indirectly to coal burning emissions from an electric-power plant [68]. A panel of oxidative stress biomarkers was measured before and after supplementation with vitamins C (500 mg) and E (800 mg) for six months and compared to a control group. In individuals exposed to PM, markers of lipid and protein damage increased and the levels of non-enzymatic antioxidants (vitamin E, GSH and protein thiols) decreased. The activities of several enzymes involved in the antioxidant defense system (catalase, glutathione peroxidase, glutathione reductase and glutathione s-transferase) were impaired, however, the activity of SOD was increased. Together, this data suggests increased utilization of antioxidants and activation of the oxidative stress defense system in the presence of PM exposure. In these subjects, vitamin E and C supplementation was effective in decreasing markers of lipid and protein damage and improved both enzymatic and non-enzymatic antioxidant defenses [68]. The ability of dietary antioxidants to enhance the activity of antioxidant enzymes is vital, as these endogenous enzymes play an integral role in neutralizing the harmful effects of free radicals, such hydrogen peroxide and the superoxide radical. Thus, antioxidant supplementation may be helpful in reducing air pollution-induced oxidative stress in the body, by both direct and indirect mechanisms.

A key mechanism by which antioxidant enzyme defenses may be enhanced is via upregulation of nuclear factor E2-related factor 2 (Nrf2). Nrf2 is the transcription factor responsible for the expression of antioxidant response element (ARE)-regulated genes, and, as such, is the master regulator of the endogenous antioxidant enzyme system. One of the most potent natural ligands for Nrf2 is sulforaphane, found in cruciferous vegetables such as broccoli and brussel sprouts. A limited number of human studies have shown that broccoli extracts or sulforaphane can have protective effects against air pollution, via inducing Nrf2-regulated gene expression in the upper airway of human subjects [67], attenuating nasal allergic response to diesel exhaust particles [70] and increasing the excretion of carcinogenic air pollutants, including benzene [71]. Further investigation of Nrf2 activation as a strategy for protecting against air pollution exposure is warranted.

Air pollution may also have an indirect negative impact on vitamin D status. Vitamin D can be synthesized in the skin through the action of sunlight. This is the major source of vitamin D, covering about 80% to 90% of our needs, since vitamin D is found naturally only in a few food items. Therefore, vitamin D status in humans is mainly determined by exposure to ultraviolet B (UVB) radiation, which initiates the conversion of 7-dehydrocholesterol to vitamin D_3_. Air pollution is a key determinant of the amount of UVB radiation from the sun that reaches the earth’s surface. Manicourt *et al.* [19] investigated the relationship between vitamin D status and a sun exposure index in postmenopausal women living either in an urban or a rural environment in Belgium. As expected, vitamin D status, assessed by measuring the 25-hydroxyvitamin D [25(OH)D] serum levels, increased linearly as the index of sun exposure increased. However, to reach an optimal vitamin D status (serum 25(OH)D level of 75 nmol/L) in urban residents, the index of sun exposure was double that for rural residents [80]. This suggests that, in an urban environment, the amount of UVB reaching the earth is significantly decreased due to air pollution, which may be a neglected factor in the high prevalence of vitamin D insufficiency. The impact of air pollution on vitamin D status has only been evaluated in a few studies in large cities. In Delhi, the vitamin D status was measured in children living either in an area affected by a high level of atmospheric pollution, or in a less polluted area of the city [81]. The mean serum 25(OH)D levels of children living in the highly polluted area of Delhi was significantly lower by 54% as compared to the level in children living in a less polluted area of the city. In healthy women in Tehran, the vitamin D status was significantly lower than in women form Ghazvinian, a less polluted city in Iran, again suggesting that living in a polluted area plays a significant role in the risk of vitamin D deficiency [82]. Moreover, in modern urban societies, humans tend to spend less time participating in outdoor activities, especially in heavily polluted areas, which further limits the amount of vitamin D that can be synthesized through exposure of the skin to sunlight. Vitamin D deficiency can promote multiple diseases, particularly osteoporosis but also cardiovascular disease, diabetes and cancer [83]. Therefore, dietary intake of additional vitamin D through foods (e.g., oily fish, dairy products, egg yolk, *etc.*) or supplements may be necessary to maintain healthy vitamin D levels in large cities affected by air pollution.

The possible adverse effects of the continuous administration of vitamin supplementation on health outcomes should also be carefully evaluated and considered. The Alpha-tocopherol, ß-carotene Cancer Prevention (ATBC) and the Carotene and Retinol Efficiency Trial (CARET) found increased risk of lung cancer with the use of low dose ß‑carotene in heavy smokers and drinkers [84,85]. However, no cancer increase was seen in moderate smokers and nonsmokers. It was proposed that the combination of alcohol abuse and heavy smoking increased the free radicals in the lung tissue, which oxidized ß-carotene and increased its carcinogenic potential as an oxidant. Furthermore, the lung has high oxygen tension that lowers the activity of ß-carotene by oxidizing it. However, a postintervention study of the ATBC trial did not show an increase in the incidence of cancer [86]. Both above studies have been criticized because of the low dose of ß-carotene used (15 mg/day), since the reported anticancer benefits postulate a dose of > 30 mg/day. In another trial performed in Linxian province, China, the effects of an antioxidant mixture containing vitamin E, ß-carotene and selenium were examined on the prevention of gastric cancer. In contrast to the ATBC and CARET studies, the highest anticancer effect of the antioxidant mixture was observed in smokers [87].

## 5. The Asthma Case

Asthma affects approximately 300 million people worldwide [88]. The economic costs of asthma rank amongst the highest for chronic diseases due to the significant healthcare utilization [88]. There are multiple environmental factors that contribute to the development and progression of the disease, including exposure to allergens, viruses and air pollution. Exposure to these triggers leads to chronic inflammation and oxidative stress in the airways [10], which results in reduced lung function, symptoms including breathlessness, coughing and wheezing and can lead to acute disease exacerbations. As the respiratory tract is the portal of entry of air pollutants into the body, the lung is the first organ affected, with a range of respiratory diseases being caused or worsened by air pollution exposure, including asthma. In asthma, exposure to triggers can also lead to systemic inflammation, which can contribute to the development of co-morbidities, such as cardiovascular disease [89].

Prevention of exacerbations is a priority of asthma management. The clinical course of asthma includes acute episodic deterioration (exacerbations) with increased symptom severity and reduced lung function, which can be life threatening in some cases [90]. Air pollution is estimated to be responsible for approximately 15% of asthma exacerbations [91]. Medical practice guidelines for asthma stipulate that a key goal of treatment is to prevent exacerbations, as exacerbations pose the greatest risk to patients, cause most anxiety to patients and their families, cause the greatest stress to health care providers and generate the greatest cost to the health care system [90]. Inhaled glucocorticoids are most commonly used to maintain asthma control and reduce exacerbation risk. However, considering the costs, side effects and non-compliance issues associated with corticosteroid use, non-pharmacological interventions to prevent exacerbations are needed to reduce the burden of asthma.

Westernized diets are characterized by a low intake of fruit, vegetables, wholegrain and fish, and an increased intake of processed foods, resulting in a nutrient intake that is low in beneficial nutrients such as antioxidants (e.g., carotenoids, ascorbic acid, tocopherols and flavonoids) and omega-3 PUFA [92]. This reduces protection against inflammatory insults, such as air pollution. Air pollution leads to both oxidative stress and inflammation, which are pathologies underlying asthma and exacerbations of asthma. Hence, increasing the intake of nutrients with antioxidant and/or anti-inflammatory properties has the potential to improve asthma management.

Antioxidants may be important in protecting the airways against air pollution; it has been reported that oxidative stress is elevated [93] and dietary antioxidant levels (carotenoids, vitamin E and C) are low in asthma [9,94,95] and correction of antioxidant deficiency, in particular lycopene (a phytochemical found in tomatoes), protects against airway neutrophilia, the type of airway inflammation that is associated with air pollution [96]. A limited number of trials have used vitamin E and C in combination to protect against the acute effects of ozone in asthma, with the largest of these studies showing an attenuation in ozone-associated lung function decline in children [64] and another trial showing protection against ozone-induced bronchial hyperresponsiveness [63]. However, supplementation with antioxidants, such as vitamin C, E and carotenoids, to mitigate the clinical effects of chronic air pollution exposure in asthma, including risk of asthma exacerbations, has not yet been studied [97]. A whole food intervention trial has been recently conducted in adults with asthma, in which fruit and vegetable intake was manipulated, as a strategy for modulating antioxidant intake [11]. A high fruit and vegetable diet led to an improvement in micronutrient status (vitamin C, E, carotenoids and B group vitamins), corresponding with a reduced risk of asthma exacerbation [11].

Omega-3 PUFA have been linked to reduced asthma risk in observational studies and preclinical studies show that omega-3 fatty acids are protective against asthma triggers, including viruses [98] and allergens [99]. However, no interventional studies have examined the effect of omega-3 fatty acids on asthma exacerbation risk [100]. Importantly, it should be noted that supplementation with omega-3 PUFA increases susceptibility of the host to oxidative damage [101]. Thus, supplementation with omega-3 PUFA in combination with antioxidants is optimal.

As previously described, vitamin D status may also be impacted by air pollution, which can prevent UV radiation from penetrating the atmosphere, resulting in reduced endogenous synthesis of vitamin D. A recent intervention trial has been conducted using vitamin D in asthma, which found that rate of first exacerbation was reduced in subjects who demonstrated an increase in circulating vitamin D_3_ following supplementation [102], suggesting the potential for vitamin D to protect against air pollution-induced exacerbations.

The evidence to date highlights the importance of intervening with relevant nutrient combinations in order to provide a protective effect against air pollution. A combined approach to nutritional intervention is logical, as nutrients are consumed in our diets in combination and work synergistically. For example, once vitamin E has performed its antioxidant function and becomes oxidized, vitamin C acts to regenerate the antioxidant form. Hence, supplementation using relevant nutrient combinations can maximize the potential benefits.

## 6. Conclusions

Air pollution is a major public health problem associated with excess cardiopulmonary morbidity and mortality worldwide. While reducing levels is the ultimate goal, achieving sustainable reductions that fully protect the population, is not likely to occur in the foreseeable future. Moving forward, air pollution related health research and policy should focus more intensively on identifying susceptibility factors, both individual and geospatial, to develop targeted interventions to reduce the ambient pollution health burden. Because both pollution composition and population variance in susceptibility is so vast, the most effective and wide-reaching interventions—beyond reductions in air pollution itself—likely operate along common inflammatory and oxidative stress pathways impacted by a range of gaseous and particulate pollutants. Interventions, which may ameliorate inflammatory effects or oxidative stress, may be among the most widely applicable.

A prudent diet is a key determinant to health throughout the whole life and could reduce the deleterious impact of air pollution on health. As demonstrated, several studies showed that some nutrients such as B vitamins, vitamin C, vitamin E, vitamin D and omega-3 PUFA have protective effects against the damage induced by PM. In an air polluted environment, a healthy diet with adequate intake of essential micronutrients may be critical to prevent the development of chronic diseases, particularly cardiovascular and pulmonary diseases. Increased intake of antioxidants, as well as other anti-inflammatory nutrients, may attenuate air-pollution induced oxidative stress and inflammation in cardiovascular disease, asthma and other chronic inflammatory diseases, thereby providing a useful addition to current disease management strategies. The potential health and economic benefits of establishing non-pharmacological approaches (e.g., dietary supplementation) to disease management are enormous. Further studies are needed to determine how various combinations of nutrients may prevent the impact of PM on different aspects of health.

## References

[1] Van Donkelaar A., Martin R.V., Brauer M., Kahn R., Levy R., Verduzco C., Villeneuve P.J. (2010). Global Estimates of Ambient Fine Particulate Matter Concentrations from Satellite-Based Aerosol Optical Depth: Development and Application. Environ. Health Perspect..

[2] WHO (2006). Who Air Quality Guidelines for Particulate Matter, Ozone, Nitrogen Dioxide and Sulfur Dioxide.

[3] Aphekom. http://www.Aphekom.Org/Web/Aphekom.Org/Home.

[4] Brook R.D. (2008). Cardiovascular Effects of Air Pollution. Clin. Sci..

[5] Peters A. (2005). Particulate Matter and Heart Disease: Evidence from Epidemiological Studies. Toxicol. Appl. Pharmacol..

[6] Romieu I., Castro-Giner F., Kunzli N., Sunyer J. (2008). Air Pollution, Oxidative Stress and Dietary Supplementation: A Review. Eur. Respir. J..

[7] Hennig B., Ettinger A.S., Jandacek R.J., Koo S., Mcclain C., Seifried H., Silverstone A., Watkins B., Suk W.A. (2007). Using Nutrition for Intervention and Prevention Against Environmental Chemical Toxicity and Associated Diseases. Environ. Health Perspect..

[8] Romieu I., Tellez-Rojo M.M., Lazo M., Manzano-Patino A., Cortez-Lugo M., Julien P., Belanger M.C., Hernandez-Avila M., Holguin F. (2005). Omega-3 Fatty Acid Prevents Heart Rate Variability Reductions Associated with Particulate Matter. Am. J. Respir. Crit. Care Med..

[9] Kelly F.J., Mudway I., Blomberg A., Frew A., Sandstrom T. (1999). Altered Lung Antioxidant Status in Patients with Mild Asthma. Lancet.

[10] Wood L.G., Gibson P.G., Garg M.L. (2003). Biomarkers of Lipid Peroxidation, Airway Inflammation and Asthma. Eur. Respir. J..

[11] Wood L.G., Garg M.L., Smart J.M., Scott H.A., Barker D., Gibson P.G. (2012). Manipulating Antioxidant Intake in Asthma: A Randomized Controlled Trial. Am. J. Clin. Nutr..

[12] Su J.G., Apte J.S., Lipsitt J., Garcia-Gonzales D.A., Beckerman B.S., de Nazelle A., Texcalac-Sangrador J.L., Jerrett M. (2015). Populations Potentially Exposed to Traffic-Related Air Pollution in Seven World Cities. Environ. Int..

[13] USEP National Ambient Air Quality Standards (Naaqs). http://www.Epa.Gov/Air/Criteria.Html.

[14] AGDOT National Standards for Criteria Air Pollutants in Australia. http://www.Environment.Gov.Au/Protection/Publications/Factsheet-National-Standards-Criteria-Air-Pollutants-Australia.

[15] Sampson P.D., Richards M., Szpiro A.A., Bergen S., Sheppard L., Larson T.V., Kaufman J.D. (2013). A Regionalized National Universal Kriging Model Using Partial Least Squares Regression for Estimating Annual Pm Concentrations in Epidemiology. Atmos. Environ..

[16] Novotny E.V., Bechle M.J., Millet D.B., Marshall J.D. (2011). National Satellite-Based Land-Use Regression: NO_2_ in the United States. Environ. Sci. Technol..

[17] Clougherty J.E., Kheirbek I., Eisl H.M., Ross Z., Pezeshki G., Gorczynski J.E., Johnson S., Markowitz S., Kass D., Matte T. (2013). Intra-Urban Spatial Variability in Wintertime Street-Level Concentrations of Multiple Combustion-Related Air Pollutants: The New York City Community Air Survey (Nyccas). J. Expo. Sci. Environ. Epidemiol..

[18] De Hoogh K., Wang M., Adam M., Badaloni C., Beelen R., Birk M., Cesaroni G., Cirach M., Declercq C., Dedele A. (2013). Development of Land Use Regression Models for Particle Composition in Twenty Study Areas in Europe. Environ. Sci. Technol..

[19] Levy J.I., Clougherty J.E., Baxter L.K., Houseman E.A., Paciorek C.J. (2010). Evaluating Heterogeneity in Indoor and Outdoor Air Pollution Using Land-Use Regression and Constrained Factor Analysis. Res. Rep..

[20] Nel A.E., Diaz-Sanchez D., Li N. (2001). The Role of Particulate Pollutants in Pulmonary Inflammation and Asthma: Evidence for the Involvement of Organic Chemicals and Oxidative Stress. Curr. Opin. Pulm. Med..

[21] Schauer J.J., Lough G.C., Shafer M.M., Christensen W.F., Arndt M.F., Deminter J.T., Park J.S. (2006). Characterization of Metals Emitted from Motor Vehicles. Res. Rep..

[22] Lanki T., de Hartog J.J., Heinrich J., Hoek G., Janssen N.A., Peters A., Stolzel M., Timonen K.L., Vallius M., Vanninen E. (2006). Can We Identify Sources of Fine Particles Responsible for Exercise-Induced Ischemia on Days with Elevated Air Pollution? The Ultra Study. Environ. Health Perspect..

[23] Clougherty Je K.I., Johnson S., Pezeshki G., Jacobson J.B., Eisl H., Gorczynski J., Ross Z., Kitson H., Benson A., Camacho A. (2010). The New York City Community Air Survey Supplemental Report: Nickel Concentrations in Ambient Fine Particles: Winter Monitoring, 2008–2009.

[24] Krewski D.B.R., Goldberg M.S., Hoover K., Siemiatycki J., Jerrett M. (2000). Reanalysis of the Harvard Six Cities Study and the American Cancer Society Study of Particulate Air Pollution and Mortality.

[25] Jerrett M., Burnett R.T., Brook J., Kanaroglou P., Giovis C., Finkelstein N., Hutchison B. (2004). Do Socioeconomic Characteristics Modify the Short Term Association between Air Pollution and Mortality? Evidence from A Zonal Time Series in Hamilton, Canada. J. Epidemiol. Commun. Health.

[26] Shmool J.L., Kubzansky L.D., Newman O.D., Spengler J., Shepard P., Clougherty J.E. (2014). Social Stressors and Air Pollution Across New York City Communities: A Spatial Approach for Assessing Correlations Among Multiple Exposures. Environ. Health A Glob. Access Sci. Sour..

[27] Brook R.D., Rajagopalan S., Pope C.A., Brook J.R., Bhatnagar A., Diez-Roux A.V., Holguin F., Hong Y., Luepker R.V., Mittleman M.A. (2010). Particulate Matter Air Pollution and Cardiovascular Disease: An Update to the Scientific Statement from the American Heart Association. Circulation.

[28] Miller M.R. (2014). The Role of Oxidative Stress in the Cardiovascular Actions of Particulate Air Pollution. Biochem. Soc. Trans..

[29] Park S.K., O’neill M.S., Wright R.O., Hu H., Vokonas P.S., Sparrow D., Suh H., Schwartz J. (2006). Hfe Genotype, Particulate Air Pollution, and Heart Rate Variability: A Gene-Environment Interaction. Circulation.

[30] Oftedal B., Brunekreef B., Nystad W., Madsen C., Walker S.E., Nafstad P. (2008). Residential Outdoor Air Pollution and Lung Function in Schoolchildren. Epidemiology.

[31] Rojas-Martinez R., Perez-Padilla R., Olaiz-Fernandez G., Mendoza-Alvarado L., Moreno-Macias H., Fortoul T., Mcdonnell W., Loomis D., Romieu I. (2007). Lung Function Growth in Children with Long-Term Exposure to Air Pollutants in Mexico City. Am. J. Respir. Crit. Care Med..

[32] Pope C.A., Burnett R.T., Thun M.J., Calle E.E., Krewski D., Ito K., Thurston G.D. (2002). Lung Cancer, Cardiopulmonary Mortality, and Long-Term Exposure to Fine Particulate Air Pollution. Jama J. Am. Med. Assoc..

[33] Raaschou-Nielsen O., Andersen Z.J., Beelen R., Samoli E., Stafoggia M., Weinmayr G., Hoffmann B., Fischer P., Nieuwenhuijsen M.J., Brunekreef B. (2013). Air Pollution and Lung Cancer Incidence in 17 European Cohorts: Prospective Analyses from the European Study of Cohorts for Air Pollution Effects (Escape). Lancet. Oncol..

[34] Ranft U., Schikowski T., Sugiri D., Krutmann J., Kramer U. (2009). Long-Term Exposure to Traffic-Related Particulate Matter Impairs Cognitive Function in the Elderly. Environ. Res..

[35] Hoek G., Brunekreef B., Goldbohm S., Fischer P., Van Den Brandt P.A. (2002). Association Between Mortality and Indicators of Traffic-Related Air Pollution in the Netherlands: A Cohort Study. Lancet.

[36] Pope C.A., Burnett R.T., Turner M.C., Cohen A., Krewski D., Jerrett M., Gapstur S.M., Thun M.J. (2011). Lung Cancer and Cardiovascular Disease Mortality Associated with Ambient Air Pollution and Cigarette Smoke: Shape of the Exposure-Response Relationships. Environ. Health Perspect..

[37] Haryanto B., Suksmasari T., Wintergerst E., Maggini S. (2015). Multivitamin Supplementation Supports Immune Function and Ameliorates Conditions Triggered By Reduced Air Quality. Vitam. Miner..

[38] Zeka A., Sullivan J.R., Vokonas P.S., Sparrow D., Schwartz J. (2006). Inflammatory Markers and Particulate Air Pollution: Characterizing the Pathway to Disease. Int. J. Epidemiol..

[39] Fujisawa T. (2005). Role of Oxygen Radicals on Bronchial Asthma. Curr. Drug Targets Inflam. Allergy.

[40] Kelly F.J., Fussell J.C. (2015). Linking Ambient Particulate Matter Pollution Effects with Oxidative Biology and Immune Responses. Ann. N. Y. Acad. Sci..

[41] Holguin F., Tellez-Rojo M.M., Hernandez M., Cortez M., Chow J.C., Watson J.G., Mannino D., Romieu I. (2003). Air Pollution and Heart Rate Variability Among the Elderly in Mexico City. Epidemiology.

[42] Sarnat J.A., Holguin F. (2007). Asthma and Air Quality. Curr. Opin. Pulm. Med..

[43] Kelly F.J., Fussell J.C. (2011). Air Pollution and Airway Disease. Clin. Exp. Allergy J. Br. Soc. Allergy Clin. Immunol..

[44] Ko F.W., Hui D.S. (2012). Air Pollution and Chronic Obstructive Pulmonary Disease. Respirology.

[45] Kunzli N., Jerrett M., Garcia-Esteban R., Basagana X., Beckermann B., Gilliland F., Medina M., Peters J., Hodis H.N., Mack W.J. (2010). Ambient Air Pollution and the Progression of Atherosclerosis in Adults. PLos ONE.

[46] Kunzli N., Jerrett M., Mack W.J., Beckerman B., Labree L., Gilliland F., Thomas D., Peters J., Hodis H.N. (2005). Ambient Air Pollution and Atherosclerosis in Los Angeles. Environ. Health Perspect..

[47] Gauderman W.J., Avol E., Gilliland F., Vora H., Thomas D., Berhane K., Mcconnell R., Kuenzli N., Lurmann F., Rappaport E. (2004). The Effect of Air Pollution on Lung Development from 10 to 18 Years of Age. N. Engl. J. Med..

[48] Mccreanor J., Cullinan P., Nieuwenhuijsen M.J., Stewart-Evans J., Malliarou E., Jarup L., Harrington R., Svartengren M., Han I.K., Ohman-Strickland P. (2007). Respiratory Effects of Exposure to Diesel Traffic in Persons with Asthma. N. Engl. J. Med..

[49] Mcconnell R., Berhane K., Yao L., Jerrett M., Lurmann F., Gilliland F., Kunzli N., Gauderman J., Avol E., Thomas D. (2006). Traffic, Susceptibility, and Childhood Asthma. Environ. Health Perspect..

[50] Holguin F., Flores S., Ross Z., Cortez M., Molina M., Molina L., Rincon C., Jerrett M., Berhane K., Granados A. (2007). Traffic-Related Exposures, Airway Function, Inflammation, and Respiratory Symptoms in Children. Am. J. Respir. Crit. Care Med..

[51] Peters A., von Klot S., Heier M., Trentinaglia I., Hormann A., Wichmann H.E., Lowel H., Cooperative Health Research in the Region of Augsburg Study Group (2004). Exposure to Traffic and the Onset of Myocardial Infarction. N. Engl. J. Med..

[52] Friedman M.S., Powell K.E., Hutwagner L., Graham L.M., Teague W.G. (2001). Impact of Changes in Transportation and Commuting Behaviors During the 1996 Summer Olympic Games in Atlanta on Air Quality and Childhood Asthma. Jama.

[53] Mu L., Deng F., Tian L., Li Y., Swanson M., Ying J., Browne R.W., Rittenhouse-Olson K., Zhang J.J., Zhang Z.F. (2014). Peak Expiratory Flow, Breath Rate and Blood Pressure in Adults with Changes in Particulate Matter Air Pollution During the Beijing Olympics: A Panel Study. Environ. Res..

[54] Zhang J., Zhu T., Kipen H., Wang G., Huang W., Rich D., Zhu P., Wang Y., Lu S.E., Ohman-Strickland P. (2013). Cardiorespiratory Biomarker Responses in Healthy Young Adults to Drastic Air Quality Changes Surrounding the 2008 Beijing Olympics. Res. Rep..

[55] Chiuve S.E., Sampson L., Willett W.C. (2011). The Association Between A Nutritional Quality Index and Risk of Chronic Disease. Am. J. Prev. Med..

[56] Hoeft B., Weber P., Eggersdorfer M. (2012). Micronutrients—A Global Perspective on Intake, Health Benefits and Economics. Int. J. Vitam. Nutr. Res..

[57] Landete J.M. (2013). Dietary Intake of Natural Antioxidants: Vitamins and Polyphenols. Crit. Rev. Food Sci. Nutr..

[58] Prasad S., Sung B., Aggarwal B.B. (2012). Age-Associated Chronic Diseases Require Age-Old Medicine: Role of Chronic Inflammation. Prevent. Med..

[59] Poljsak B., Fink R. (2014). The Protective Role of Antioxidants in the Defence Against Ros/Rns-Mediated Environmental Pollution. Oxid. Med. Cell. Longev..

[60] Mohsenin V. (1987). Effect of Vitamin C on No2-Induced Airway Hyperresponsiveness in Normal Subjects. A Randomized Double-Blind Experiment. Am. Rev. Respir. Dis..

[61] Romieu I., Meneses F., Ramirez M., Ruiz S., Perez Padilla R., Sienra J.J., Gerber M., Grievink L., Dekker R., Walda I. (1998). Antioxidant Supplementation and Respiratory Functions Among Workers Exposed to High Levels of Ozone. Am. J. Respir. Crit. Care Med..

[62] Grievink L., Zijlstra A.G., Ke X., Brunekreef B. (1999). Double-Blind Intervention Trial on Modulation of Ozone Effects on Pulmonary Function By Antioxidant Supplements. Am. J. Epidemiol..

[63] Trenga C.A., Koenig J.Q., Williams P.V. (2001). Dietary Antioxidants and Ozone-Induced Bronchial Hyperresponsiveness in Adults with Asthma. Arch. Environ. Health.

[64] Romieu I., Sienra-Monge J.J., Ramirez-Aguilar M., Tellez-Rojo M.M., Moreno-Macias H., Reyes-Ruiz N.I., Del Rio-Navarro B.E., Ruiz-Navarro M.X., Hatch G., Slade R. (2002). Antioxidant Supplementation and Lung Functions Among Children with Asthma Exposed to High Levels of Air Pollutants. Am. J. Respir. Crit. Care Med..

[65] Sienra-Monge J.J., Ramirez-Aguilar M., Moreno-Macias H., Reyes-Ruiz N.I., Del Rio-Navarro B.E., Ruiz-Navarro M.X., Hatch G., Crissman K., Slade R., Devlin R.B. (2004). Antioxidant Supplementation and Nasal Inflammatory Responses Among Young Asthmatics Exposed to High Levels of Ozone. Clin. Exp. Immunol..

[66] Romieu I., Garcia-Esteban R., Sunyer J., Rios C., Alcaraz-Zubeldia M., Velasco S.R., Holguin F. (2008). The Effect of Supplementation with Omega-3 Polyunsaturated Fatty Acids on Markers of Oxidative Stress in Elderly Exposed to Pm (2.5). Environ. Health Perspect..

[67] Riedl M.A., Saxon A., Diaz-Sanchez D. (2009). Oral Sulforaphane Increases Phase Ii Antioxidant Enzymes in the Human Upper Airway. Clin. Immunol..

[68] Possamai F.P., Junior S.A., Parisotto E.B., Moratelli A.M., Inacio D.B., Garlet T.R., dal-Pizzol F., Filho D.W. (2010). Antioxidant Intervention Compensates Oxidative Stress in Blood of Subjects Exposed to Emissions from A Coal Electric-Power Plant in South Brazil. Environ. Toxicol Pharmacol..

[69] Tong H., Rappold A.G., Diaz-Sanchez D., Steck S.E., Berntsen J., Cascio W.E., Devlin R.B., Samet J.M. (2012). Omega-3 Fatty Acid Supplementation Appears to Attenuate Particulate Air Pollution-Induced Cardiac Effects and Lipid Changes in Healthy Middle-Aged Adults. Environ. Health Perspect..

[70] Heber D., Li Z., Garcia-Lloret M., Wong A.M., Lee T.Y., Thames G., Krak M., Zhang Y., Nel A. (2014). Sulforaphane-Rich Broccoli Sprout Extract Attenuates Nasal Allergic Response to Diesel Exhaust Particles. Food Funct..

[71] Egner P.A., Chen J.G., Zarth A.T., Ng D., Wang J., Kensler K.H., Jacobson L.P., Munoz A., Johnson J.L., Groopman J.D. (2014). Rapid and Sustainable Detoxication of Airborne Pollutants By Broccoli Sprout Beverage: Results of A Randomized Clinical Trial in China. Cancer Prev. Res..

[72] Lavie C.J., Milani R.V., Mehra M.R., Ventura H.O. (2009). Omega-3 Polyunsaturated Fatty Acids and Cardiovascular Diseases. J. Am. Coll. Cardiol..

[73] Harris W.S., Kris-Etherton P.M., Harris K.A. (2008). Intakes of Long-Chain Omega-3 Fatty Acid Associated with Reduced Risk for Death from Coronary Heart Disease in Healthy Adults. Curr. Atheroscler. Rep..

[74] Mozaffarian D., Geelen A., Brouwer I.A., Geleijnse J.M., Zock P.L., Katan M.B. (2005). Effect of Fish Oil on Heart Rate in Humans: A Meta-Analysis of Randomized Controlled Trials. Circulation.

[75] Kelly F.J., Mudway I.S. (2003). Protein Oxidation At the Air-Lung Interface. Amino Acids.

[76] Kelly F.J. (2004). Dietary Antioxidants and Environmental Stress. Proc. Nutr. Soc..

[77] Fiorito G., Guarrera S., Valle C., Ricceri F., Russo A., Grioni S., Mattiello A., di Gaetano C., Rosa F., Modica F. (2014). B-Vitamins Intake, Dna-Methylation of One Carbon Metabolism and Homocysteine Pathway Genes and Myocardial Infarction Risk: The Epicor Study. Nutr. Metab. Cardiovasc. Dis. Nmcd.

[78] Abbenhardt C., Miller J.W., Song X., Brown E.C., Cheng T.Y., Wener M.H., Zheng Y., Toriola A.T., Neuhouser M.L., Beresford S.A. (2014). Biomarkers of One-Carbon Metabolism Are Associated with Biomarkers of Inflammation in Women. J. Nutr..

[79] Baccarelli A., Cassano P.A., Litonjua A., Park S.K., Suh H., Sparrow D., Vokonas P., Schwartz J. (2008). Cardiac Autonomic Dysfunction: Effects from Particulate Air Pollution and Protection By Dietary Methyl Nutrients and Metabolic Polymorphisms. Circulation.

[80] Manicourt D.H., Devogelaer J.P. (2008). Urban Tropospheric Ozone Increases the Prevalence of Vitamin D Deficiency Among Belgian Postmenopausal Women with Outdoor Activities During Summer. J. Clin. Endocrinol. Metab..

[81] Agarwal K.S., Mughal M.Z., Upadhyay P., Berry J.L., Mawer E.B., Puliyel J.M. (2002). The Impact of Atmospheric Pollution on Vitamin D Status of Infants and Toddlers in Delhi, India. Arch. Dis. Child..

[82] Hosseinpanah F., Pour S.H., Heibatollahi M., Moghbel N., Asefzade S., Azizi F. (2010). The Effects of Air Pollution on Vitamin D Status in Healthy Women: A Cross Sectional Study. BMC Public Health.

[83] Holick M.F. (2011). Vitamin D Deficiency in 2010: Health Benefits of Vitamin D and Sunlight: A D-Bate. Nat. Rev. Endocrinol..

[84] Albanes D., Heinonen O.P., Taylor P.R., Virtamo J., Edwards B.K., Rautalahti M., Hartman A.M., Palmgren J., Freedman L.S., Haapakoski J. (1996). Alpha-Tocopherol and Beta-Carotene Supplements and Lung Cancer Incidence in the Alpha-Tocopherol, Beta-Carotene Cancer Prevention Study: Effects of Base-Line Characteristics and Study Compliance. J. Natl. Cancer Inst..

[85] Omenn G.S., Goodman G.E., Thornquist M.D., Balmes J., Cullen M.R., Glass A., Keogh J.P., Meyskens F.L., Valanis B., Williams J.H. (1996). Risk Factors for Lung Cancer and for Intervention Effects in Caret, the Beta-Carotene and Retinol Efficacy Trial. J. Natl. Cancer Inst..

[86] Virtamo J., Pietinen P., Huttunen J.K., Korhonen P., Malila N., Virtanen M.J., Albanes D., Taylor P.R., Albert P., Group A.S. (2003). Incidence of Cancer and Mortality Following Alpha-Tocopherol and Beta-Carotene Supplementation: A Postintervention Follow-Up. Jama.

[87] Blaylock R.L. (2015). Methodological Problems with Population Cancer Studies: The Forgotten Confounding Factors. Surg. Neurol. Int..

[88] Masoli M., Fabian D., Holt S., Beasley R. (2004). Global Initiative for Asthma (Gina) Program. The Global Burden of Asthma: Executive Summary of the Gina Dissemination Committee Report. Allergy.

[89] Wood L.G., Baines K.J., Fu J., Scott H.A., Gibson P.G. (2012). The Neutrophilic Inflammatory Phenotype Is Associated with Systemic Inflammation in Asthma. Chest.

[90] Reddel H.K., Taylor D.R., Bateman E.D., Boulet L.P., Boushey H.A., Busse W.W., Casale T.B., Chanez P., Enright P.L., Gibson P.G. (2009). An Official American Thoracic Society/European Respiratory Society Statement: Asthma Control and Exacerbations: Standardizing Endpoints for Clinical Asthma Trials and Clinical Practice. Am. J. Respir. Crit. Care Med..

[91] European Respiratory Society Ers White Book. http://www.Erswhitebook.Org/Chapters/Outdoor-Environment/.

[92] Troesch B., Hoeft B., Mcburney M., Eggersdorfer M., Weber P. (2012). Dietary Surveys Indicate Vitamin Intakes Below Recommendations Are Common in Representative Western Countries. Br. J. Nutr..

[93] Wood L.G., Garg M.L., Simpson J.L., Mori T.A., Croft K.D., Wark P.A., Gibson P.G. (2005). Induced Sputum 8-Isoprostane Concentrations in Inflammatory Airway Diseases. Am. J. Respir. Crit. Care Med..

[94] Wood L.G., Garg M.L., Blake R.J., Garcia-Caraballo S., Gibson P.G. (2005). Airway and Circulating Levels of Carotenoids in Asthma and Healthy Controls. J. Am. Coll. Nutr..

[95] Wood L.G., Garg M.L., Blake R.J., Simpson J.L., Gibson P.G. (2008). Oxidized Vitamin E and Glutathione As Markers of Clinical Status in Asthma. Clin. Nutr..

[96] Wood L.G., Garg M.L., Powell H., Gibson P.G. (2008). Lycopene-Rich Treatments Modify Noneosinophilic Airway Inflammation in Asthma: Proof of Concept. Free Radic. Res..

[97] Wilkinson M., Hart A., Milan S.J., Sugumar K. (2014). Vitamins C and E for Asthma and Exercise-Induced Bronchoconstriction. Cochrane Database Syst. Rev..

[98] Saedisomeolia A., Wood L.G., Garg M.L., Gibson P.G., Wark P.A. (2009). Anti-Inflammatory Effects of Long-Chain N-3 Pufa in Rhinovirus-Infected Cultured Airway Epithelial Cells. Br. J. Nutr..

[99] Wood L.G., Hazlewood L.C., Foster P.S., Hansbro P.M. (2010). Lyprinol Reduces Inflammation and Improves Lung Function in A Mouse Model of Allergic Airways Disease. Clin. Exp. Allergy.

[100] Thien F.C., Woods R.K., Abramson M.J. (2004). Dietary Marine Fatty Acids (Fish Oil) for Asthma in Adults and Children (Cochrane Review). Cochrane Librar..

[101] Saedisomeolia A., Wood L.G., Garg M.L., Gibson P.G., Wark P.A.B. (2008). Supplementation of Long Chain *N*-3 Polyunsaturated Fatty Acids Increases Utilisation of Lycopene in Cultured Airway Epithelial Cells. J. Food Lipids.

[102] Castro M., King T.S., Kunselman S.J., Cabana M.D., Denlinger L., Holguin F., Kazani S.D., Moore W.C., Moy J., Sorkness C.A. (2014). Effect of Vitamin D3 on Asthma Treatment Failures in Adults with Symptomatic Asthma and Lower Vitamin D Levels: The Vida Randomized Clinical Trial. Jama.

